# Loss of Sunda clouded leopards and forest integrity drive potential impacts of mesopredator release on vulnerable avifauna

**DOI:** 10.1016/j.heliyon.2024.e32801

**Published:** 2024-06-11

**Authors:** Darwin S. Mayhew, Andrew J. Hearn, Olivier Devineau, John D.C. Linnell, David W. Macdonald

**Affiliations:** a Department of Forestry and Wildlife Management, Inland Norway University of Applied Sciences - Campus Evenstad, Anne Evenstads Vei 80, 2480, Koppang, Norway; b Norwegian Institute for Nature Research, Vormstuguveien 40, 2624, Lillehammer, Norway; c WildCRU, Department of Biology, University of Oxford, Recanati-Kaplan Centre, Tubney House, Abingdon Rd, Tubney, OX13 5QL, United Kingdom

**Keywords:** Forest integrity, Mesopredator release, Bird, Structural equation model, Trophic cascade, Oil palm plantations

## Abstract

Amongst the unintended consequences of anthropogenic landscape conversion is declining apex predator abundance linked to loss of forest integrity, which can potentially re-order trophic networks. One such re-ordering, known as mesopredator release, occurs when medium-sized predators, also called mesopredators, rapidly increase in abundance following the decline in apex predator abundance, consequently reducing the abundance of mesopredator prey, notably including terrestrial avifauna. We examine the cascading impacts of declining Sunda clouded leopard abundance, itself consequent upon a reduction in forest integrity, on the mesopredator community of Sabah, Malaysia, to determine whether the phenomenon of mesopredator release is manifest and specifically whether it impacts the terrestrial avifauna community of pheasants and pittas. To explore this trophic interaction, we used a piecewise structural equation model to compare changes in the relative abundance of organisms. Our results suggest that loss of forest integrity may have broad impacts on the community and trigger mesopredator release, the two acting additively in their impact on already vulnerable species of terrestrial avifauna: a result not previously documented in tropical systems and rarely detected even on a global scale. The limiting effect that the Sunda clouded leopard has on the Sunda leopard cat could illuminate the mechanism whereby mesopredator release impacts this system. Both Bulwer's pheasant and pittas appear to be significantly impacted by the increase in Sunda leopard cats, while the great argus pheasant shows similar compelling, although not statistically significant, declines as Sunda leopard cats increase. The inverse relationship between Sunda clouded leopards and Sunda leopard cats suggests that if a mesopredator release exists it could have downstream consequences for some terrestrial avifauna. These results suggest the under-studied interface between mammalian carnivores and avifauna, or more broadly species interactions in general, could offer important conservation tool for holistic ecosystem conservation efforts.

## Highlights

• The first study to show mesopredator release impacts on tropical terrestrial birds.
• Threatened pheasants may be affected by terrestrial mesopredator release mechanics.
• Structural equation modeling can create detailed ecological interaction networks.
• Applies forest integrity in SEMs as a measure of abiotic and biotic factors.
• Camera trapping data is useful in evaluating carnivore and avifauna relationships.

## 1 Introduction

The dynamics of ecological communities in forests remain poorly understood, with cryptic species being amongst those lost at the highest rates to deforestation or agricultural conversion in tropical forest systems [1,2]. The ecological impacts of degraded forest integrity, and their consequences, have become critical to understanding biodiversity loss in ecological networks [3,4]. Forest integrity loss not only has direct impacts at varying trophic levels but can also result in cascading impacts between trophic levels [5]. Apex predators are of particular interest in this context as their disappearance, often triggered by external effects such as landscape conversion, can lead to trophic cascades through the food web that can alter fundamental ecosystem functions [[6], [7], [8]].

One mechanism by which these consequences can occur is mesopredator release, in which the decline in, or disappearance of, an apex predator's population results in a population increase of small to medium-sized predators (mesopredators) which in turn causes a decline in populations of the latter's prey [[9], [10], [11], [12]]. This mechanism is considered to occur at higher frequencies when mesopredators utilize prey also utilized by the apex predator and when the body-mass ration of apex predators to mesopredators is on average between 2 and 5.4 as to ensure the risk-reward trade-off of interspecific killing is in the apex predators favor [11]. While mesopredator release has been thoroughly documented in intraclass systems, it can also impact prey at the intersection of terrestrial and avian communities through nest depredation, direct predation, or a combination of the two [13,14]. An early documentation of this effect of mesopredator release revealed how fragmentation of the Southern Californian landscapes led to a decline in coyotes (*Canis latrans*) causing a mesopredator release of domesticated cats (*Felis catus*) that had cascading impacts on their avian prey [13].

As extinction rates amongst rare, specialized, and large-bodied species of tropical forest-dwelling birds are disproportionately high, mesopredator release may be a causal link between similar declines in tropical apex predators and a reduction in terrestrial forest-dwelling birds [[15], [16], [17], [18]]. The loss of birds is of concern as their decline can have various impacts on ecosystem services including changes to seed dispersal, pollination, carrion consumption, nutrient cycling, and populations of invertebrates or vertebrates, of which some are relevant as pests [19]. Globally the loss of native birds has been attributed to, among other factors, increases in domestic cat (*Felis catus*) abundance which we believe could be analogous to the effects from mesopredator release of small native felids on tropical bird species [20,21].

To explore mesopredator release at the intersection of the tropical bird and mammal communities, we investigated the impact of declining forest integrity on the vertebrate community dynamics of Sabah, Malaysia. As one of the most biodiverse places in the world, it has experienced rapid conversion of primary tropical forest to oil palm plantations that has broadly impacted forest integrity and biodiversity, including the loss of apex predators, notably the Sunda clouded leopard (*Neofelis diardi*), thereby creating conditions likely to prompt mesopredator release [4,22,23]. Previous studies have, using camera traps, primarily investigated population sizes, movement, and demographics of feline carnivores with emphasis on the Sunda clouded leopard. Unfortunately, detailed dietary and interaction data of these species is limited due to the climate of the region and challenge of capturing individuals making grounded claims about individual predation between species difficult.

Luckly, camera-trapping has proven an effective means of simultaneously monitoring some groups of terrestrial birds, such as pheasants, in addition to medium-sized and large mammals [24]. This facilitates an investigation of mesopredator release through the following species relevant to our hypothesis: Sunda clouded leopard (12.0–25.2 kg); [apex predator], Sunda leopard cat (*Prionailurus javanensis*; 1.7–2.9 kg); [mesopredator], great argus pheasant (*Argusianus argus*; 1.59–1.7 kg); [prey], Bulwer's pheasant (*Lophura bulweri*; 0.91–1.8 kg); [prey], crested fireback pheasant (*Lophura ignita*; 1.6–2.6 kg); [prey], and the pitta family consisting of six species (Family: Pittidae; 0.042–0.21 kg; specific species included: black-crowned pitta (*Erythropitta ussheri*), Bornean banded-pitta (*Hydrornis schwaneri*), blue-banded pitta (*Erythropitta arquata*), blue-headed pitta (*Hydrornis baudii*), Western hooded pitta (*Pitta sordida*), and giant pitta (*Hydrornis caeruleus*)); [prey]. Hereafter we refer to Sunda clouded leopards as clouded leopards and Sunda leopard cats as leopard cats, but these should not be confused with the mainland species of *Neofelis nebulosa* and *Prionailurus bengalensis* respectively.

Our goal is to better understand the intraguild interaction of the two felid species, and how those dynamics might impact avifauna through the cascading effects of possible mesopredator release in this relatively undocumented ecosystem. To do this we predicted 1) loss of forest integrity would be associated with decreased abundance of apex predators, 2) decrease in the abundance of apex predators would be associated with increased abundance of mesopredators suggesting mesopredator release, 3) an increase in mesopredator abundance would be associated with a subsequent decline in pheasant and pitta abundance.

## 2 Methods

### 2.1 Study design

Across the Malaysian state of Sabah on the island of Borneo camera traps were deployed between May 2007 and December 2021, with all but one camera grid initiated before January 2014. The cameras were located along roads, game trails, or ridgelines, between 0 and 1600 m in elevation at approximately regular 1-km intervals to form camera grids [[25], [26], [27]]. A total of eleven grids consisted of 15–79 camera stations totaling 498 independent stations (Table A1). At each station, two cameras were deployed (Totaling 996 cameras deployed) ca. 30 cm off the ground, facing one another, to capture both sides of photographed animals and to increase probability of detection. Six grids were in relatively intact lowland or lower montane forest. Two grids were placed in a mix of selectively logged lowland forest, fragmented plantations, and mangroves, and three in oil palm plantations [26]. However, as detection rates of our target species are non-uniform across study areas, the subdivision of the original grids helped account for the potential spatial variation in abundance and increase the strength of the general linear models that compose our subsequent piecewise structural equation model. Therefore, we used a constrained k-means clustering algorithm to subdivide the eleven grids into thirty sub-grids, each containing at least fifteen camera traps [28]. Four camera locations were removed post subdivision as they were stolen or broken while in the field resulting in no data being collected.

### 2.2 Estimation of forest integrity

**Fig. 1:**
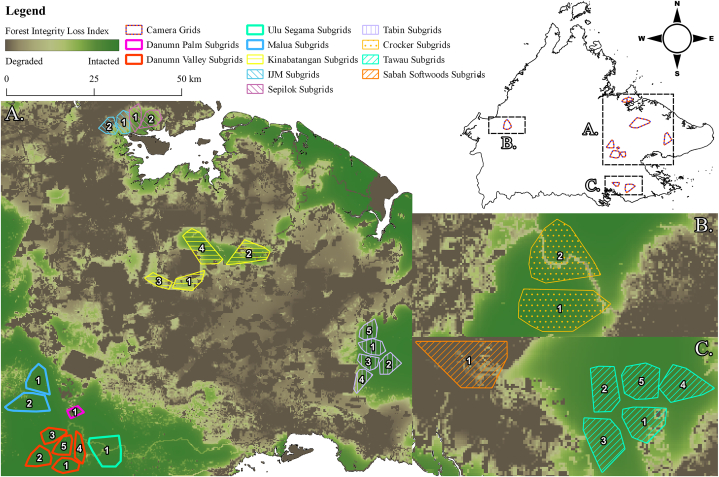
Camera Trapping Locations Camera-trapping grids are depicted as orange and blue dashed outlines in the full Sabah map. Full sub-grids are indicated by color with numbers indicating the sub-grid number in all the full grids included in A.: 1. Danum Palm, Danum Valley, IJM, Kinabatangan, Malua, Sepilok, Ulu Segama Tabin; B: Crocker; and C: Tawau, Sabah Softwoods. Forest integrity was based on the Grantham et al. [29] map of anthropogenic modification of forests with green areas representing the most intact habitat and brown areas the most degraded. Numbers indicate the sub-grid number within each color-coded grid ranging from 1 to 5 sub-grids (e.g. Danumn Valley) with some grids only having one sub-grid or in other words an unbroken original grid (e.g. Ulu Segama). (For interpretation of the references to color in this figure legend, the reader is referred to the Web version of this article.)

To represent the anthropogenic impacts of land use change, we used Grantham et al.‘s [29] forest loss integrity index, hereafter referred to as forest integrity, which is a measure of deviation from the natural state calculated for forest conditions in 2019. After visually comparing the forest integrity GIS layer to satellite imagery from data collection years, we concluded that land use had not changed significantly at the spatial scale we considered for the estimation of species abundances. Average forest integrity values were calculated for each sub-grid area (see Table A2), which was determined by calculating a minimum convex polygon (MCP) around each set of camera-trap stations that make up their respective sub-grid, plus an additional 100-m buffer added to each MCP (see Fig. 1) in QGIS [30]. However, as forest integrity was specified to range from 0 (low) to 10 (high), missing forest integrity values (as calculated by Grantham et al. [29]) were truncated to zero. This was done because missing forest integrity cell values in the original raster file were based on forest cover of less than 5 m in height; we interpreted this as equivalent to the poorest habitat possible for forest-dependent species.

### 2.3 Detectability-corrected abundance estimates

Following Cunningham et al. [31], we calculated detectability-corrected estimates of relative abundance derived from presence-absence and group counts to be incorporated into the next step of our analysis. We first used the camtrapR package [32] in the R environment [33] to collapse photographic observations of all species into 60-min intervals to ensure both temporal independence of observations and to match older camera data to newer data resulting in 57,283 useable photo records. Given the complex nature of Sabahan ecosystems and to ensure estimates were sufficiently robust, we limited our analyses to ecologically relevant species with enough camera observations to derive abundance estimates and pooled all six pitta species into a family group. We then created detection histories for each camera-trap location (N = 498) to facilitate species abundance models.

Since carnivores (clouded leopard and leopard cat) and pittas were mostly observed as single individuals, we considered these data to be presence/absence, and we used the Royle-Nichols abundance model [34] within the unmarked package [35] to estimate their abundance. On the other hand, pheasants (great argus, crested fireback, and Bulwer's pheasant) were observed in groups of varying sizes, leading us to estimate abundance using the N-mixture model [36] also within the unmarked package [35], with group size defined by the largest number of individuals observed in any one photo within the 60-min interval.

Both the Royle-Nichols model [34] and the N-mixture model [36] include a sub-model for detection, and a (latent) sub-model for abundance (see Cunningham et al. [31]; Fiske & Chandler [35]; Nakashima [37]; Royle [36]; Royle & Nichols [34] for more details on these models). We modeled detection as a function of effort (i.e., number of days a given camera station was active), and of presence/absence of forest roads and ridge lines at the individual camera station [34] (see Table A3). We modeled abundance as a function of our specific spatial sample units (i.e., 30 sub-grids) which was derived from splitting the original eleven study sites. These models produce an estimate of abundance for each of the 30 sub-grids by either exploiting the link between detection probability and abundance as with the Royle-Nichols model [34] or by using repeated count data as with the N-mixture model [36]. We only considered the 3 detection covariates listed to avoid the strong assumptions with respect to fine scale landscape change inherent to deriving more covariates from 10-years old remote sensing data under dense canopy cover of our study area [26,32,38].

### 2.4 Piecewise structural equation model

**Fig. 2:**
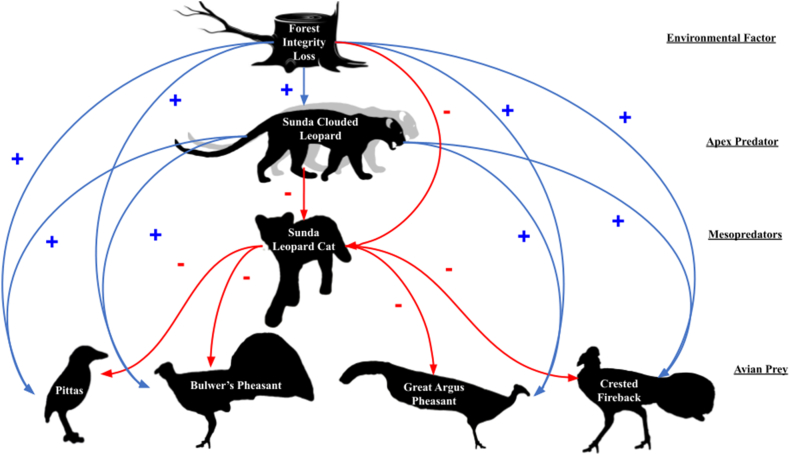
An *a-priori* piecewise SEM Forest integrity has declined rapidly due to oil palm plantations and related human influences across Southeast Asia. This figure depicts our *a-priori* piecewise structural equation model and the potential restructuring of the Sabah felid community following forest integrity loss and its subsequent effects on the terrestrial avifauna species community. Red lines indicate hypothetical negative relationships, blue lines indicate hypothetical positive relationships, and drop shadows represent predicted correlated error with a species own squared values. (For interpretation of the references to color in this figure legend, the reader is referred to the Web version of this article.)

To assess potential cascading effects in the trophic network in relation to forest integrity, we built a regression model for each of the 6 focal species. We based our focal species models on abundance estimates for each sub-grid from the previous models (N = 30), which we combined into a piecewise structural equation model (SEM) fitted with the piecewiseSEM package in R [39]. Prior to comparing abundance estimates we multiplied all numeric variables by one hundred and rounded the results to allow for the use of negative binomial models without altering the estimates. For all species we used generalized linear models with a negative binomial distribution. Owing to the limited number of sub-grids, we did not have sufficient observations to include the original camera grids as a varying intercept to account for the structure of the original study design. Starting from an *a priori* SEM model (Fig. 2), consisting of 15 pathways, we used an AIC-based stepwise model reduction to remove insignificant pathways (α = 0.05), until only significant pathways remained with one exception (see Cunningham et al. [31] or Gordon et al. [40], for a similar approach). The retention of the relationship between leopard cats and great argus pheasants in our model was non-significant based on P-values but was retained based on a less than two-point change in deltaAIC, a visual inspection of the paired data which displayed a relationship paralleling that of other terrestrial bird species retained based on P-values, and the objective of this study being an exploratory/descriptive one [41]. We assessed the overall fit of the final SEM using Shipley's test of d-separation [42,43], which tests whether all unconnected variables are conditionally independent. To account for pathways that were not conditionally independent and unspecified in our a-priori model, we specified these relationships as partially correlated to account for the effects of covariance. These relationships included one pathway between Bulwer's pheasant abundance and pitta abundance as well as a pathway between clouded leopards abundance and the squared values of clouded leopards abundance. The inclusion of these relationships in our model was not necessary for the Fisher's C statistic to have a P > 0.05; however, their inclusion helped account for all possible relevant connections (see Table A4 for a list of independence claims from the d-separation test).

## 3 Results

### 3.1 Detectability-corrected abundance estimates

Mean forest integrity ranged from 184 (low) to 994 (high). Estimates of relative abundance at each of the 30 sub-grids obtained from the N-mixture and Royle-Nichols models ranged from 0 to 272 for clouded leopards, 18 to 1775 for leopard cats, 0 to 1393 for great argus pheasants, 0 to 302 for Bulwer's pheasants, 0 to 3005 for crested firebacks, and 0 to 131 for the pitta family. All estimates and associated standard errors are presented in Table A2.

### 3.2 Piecewise structural equation model

**Fig. 3:**
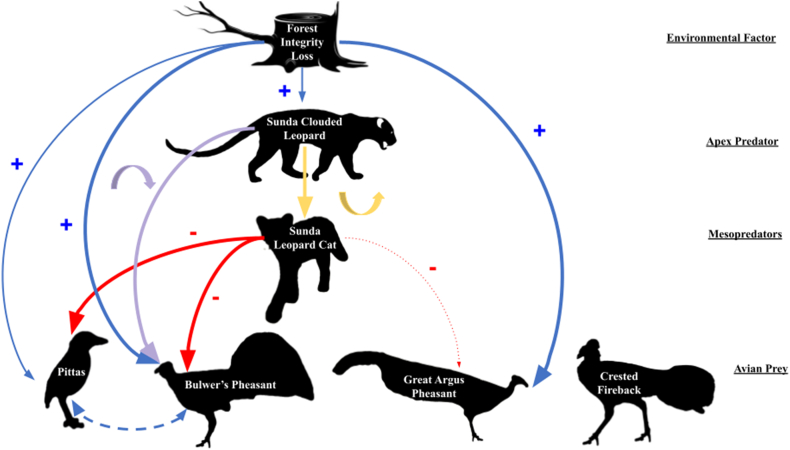
A finial piecewise SEM Our final piecewise structural equation model showing forest integrity and Sunda clouded leopard abundance have a trophic cascading effect on abundance of Sunda leopard cats and subsequently on terrestrial avifauna species. Nodes are our species of interest and the forest loss integrity index extracted from Grantham et al.‘s [29] study on global forest integrity. All solid lines represent significant pathways from our most parsimonious SEM at an alpha level of α = 0.05, with blue lines representing positive relationships, red lines representing negative relationships, purple lines representing downward parabolic relationships, and yellow lines representing upward parabolic relationships. The dotted line represents a retained non-significant pathway between Sunda leopard cats and great argus pheasants. The dashed line between pittas and Bulwer's pheasants represents a specified correlated error between variables. A second correlated error exists between Sunda clouded leopards and its own squared values that is not depicted here. Line thickness correlates with coefficient. P-values and coefficients for each species modeled in our SEM are listed in Table 1. (For interpretation of the references to color in this figure legend, the reader is referred to the Web version of this article.)

**Fig. 4:**
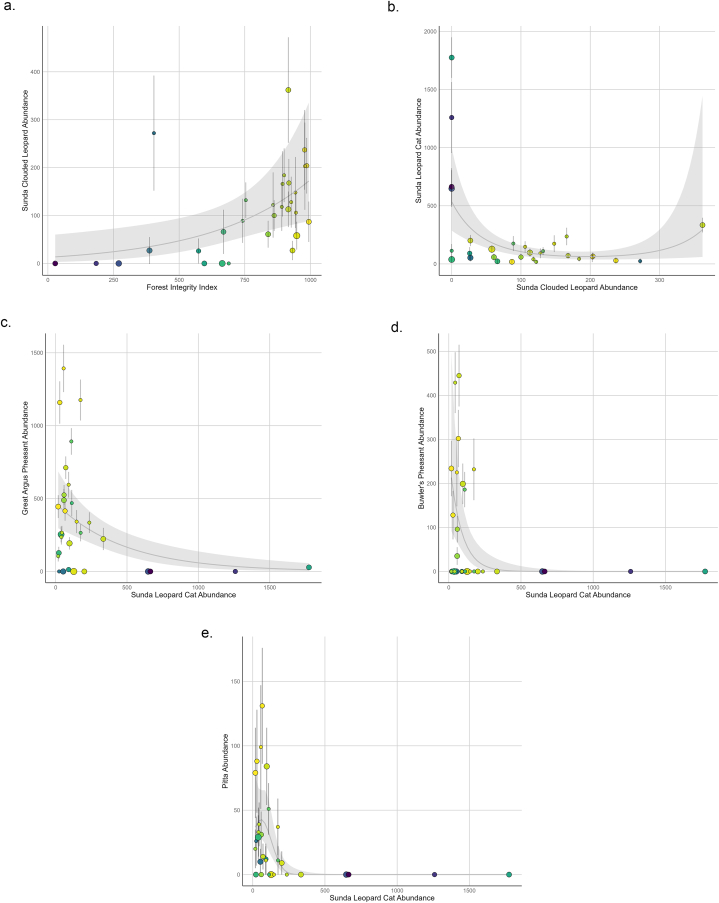
Species abundance relationship models These key pathways from our SEM depict how (a) the abundance of our apex predator the Sunda clouded leopard is tied to the forest loss integrity index and (b) how that decline subsequently results in an increase in abundance of the Sunda leopard cat, a mesopredator. In turn cascading negative impacts from increased Sunda leopard cat abundance appear to affect (c) great argus pheasant's abundance, (d) Bulwer's pheasant abundance, and (e) the pittas' abundance. Points denote detectability corrected measures of abundance for each species in each of the 30 sub-grids using the Royle-Nichols model of abundance to estimate felid species and the N-mixture model of abundance to estimate terrestrial avifauna. Each graph has a grey line (±95 % CI) indicating the respective general linear model used for each species or functional group. Forest integrity is indicated for each point on a scale from low integrity (Blue) to high integrity (Yellow). The size of the points is representative of the number of cameras in each sub-grid location with the smallest points representing the minimum camera number of fourteen and the largest a maximum of twenty-three. Forest integrity scale defined by Grantham et al. [29] low (≤600); medium (>600) and high integrity (≥960). (For interpretation of the references to color in this figure legend, the reader is referred to the Web version of this article.)

Our final piecewise Structural Equation Model (Fig. 3, Table 1) included eleven pathways and two partially correlated connections (Table A5). Forest integrity positively corresponded to four pathways including all species abundance estimates aside from leopard cats and crested firebacks but was the only predictor for clouded leopard abundance (Fig. 4a.). Clouded leopard abundance corresponded to two pathways including one quadratic relationship with leopard cat abundance (Fig. 4b.) and one quadratic relationship with Bulwer's pheasants. Leopard cats corresponded negatively with the abundance of three species of the terrestrial avifauna including great argus pheasant (Fig. 4c), Bulwer's pheasants (Fig. 4d), and pittas (Fig. 4e), although only the two pathways with Bulwer's pheasant and pitta abundance had significant relationships with leopard cat abundance.

**Table 1:** a

Model	Coefficient (SE)	P-value
Sunda clouded leopard; GLM		
(Intercept)	2.54 (0.78)	0.003 **
Mean Forest Integrity	0.003 (0.001)	0.01*
Sunda leopard cat; GLM		
(Intercept)	6.28 (0.32)	<2e-16 ***
Sunda clouded leopard	−0.02 (0.005)	0.0002***
I (Sunda clouded leopard^2)	0.006 (0.002)	0.002 **
Great Argus Pheasant; GLM		
(Intercept)	−0.64 (1.01)	0.54
Sunda leopard cat	−0.0007 (0.0007)	0.33
Mean Forest Integrity	0.008 (0.001)	2.62e-7 ***
Bulwer's Pheasant; GLM		
(Intercept)	−8.07 (2.27)	0.002 **
Sunda clouded leopard	0.12 (0.02)	3.77e-5 ***
I (Sunda clouded leopard^2)	−0.04 (0.008)	7.78e-5 ***
Sunda leopard cat	−0.016 (0.004)	0.0005 ***
Mean Forest Integrity	0.006 (0.003)	0.02 *
Pitta Family; GLM		
(Intercept)	1.90 (0.96)	0.06
Sunda leopard cat	−0.01 (0.003)	0.0001 ***
Mean Forest Integrity	0.003 (0.001)	0.02 *
Bulwer's Pheasant/Pitta Family, CE	0.50 (N/A)	0.0028 **
Sunda clouded leopard/^a^ (Sunda clouded leopard^2), CE	0.66 (N/A)	0.000 ***

a Piecewise structural equation model's results of the local estimates for each general linear model that compose the global model. Models were built using general linear models (GLM) and specified correlated error relationships (CE). Estimates are standardized and P-values are marked as significant at an α = 0.05.

## 4 Discussion

We provide some evidence for mesopredator release for the first time in the Bornean avifaunal community involving endemic felids, and we achieve this using, also for the first time in this system, a piecewise structural equation model. The community dynamics, and guild structure of felids in Borneo, and more widely in Southeast Asia, are very poorly understood. It is an important insight into their ecology that our results suggest an inverse parabolic relationship between abundances of the clouded leopards and leopard cats. Our findings also make a case that disruptive cascading impacts of landscape conversion to oil palm plantations change the mesopredator and ground bird community through the reduction in clouded leopard abundance as a result of a possible mesopredator release of the leopard cat.

It is highly probable that clouded leopard populations have declined over the last century, likely triggered by the decrease in forest integrity related to the increase in oil palm plantations [23]. Conversely, leopard cats are considered “oil palm adapters'', using human-dominated landscapes to avoid predation, competition from other felid species, and/or to take advantage of the heterogeneity in the landscape for food or shelter [44]. Our results add to these hypotheses by suggesting that Sunda leopard cats flourish in oil palm plantations partly because of the absence of clouded leopards, which leads to an increase in their abundance in this habitat. Notably our findings depict an inverse parabolic relationship between the abundance of clouded leopards and leopard cats but no relationship between forest integrity and leopard cat abundance which we would expect if habitat preference were driving this relationship.

This relationship between clouded leopards and leopard cats is possibly linked to our small sample size, which we suspect, given the prevailing negative relationship, would smooth out as sample size increases highlighting an overall negative relationship between the two species. In subsequent testing the removal of the outlying point driving the parabolic relationship resulted in a significant negative relationship. However, given the small sample size and lack of tools in the SEM, such as general additive models (GAM), the quadratic function was used to increase flexibility in our model to capture the non-linear relationship caused by the impact of statistical outliers and/or any potentially unintended abiotic or biotic factors we may not have included in the model. In an attempt to offer a biological explanation for the inverse parabolic relationship presented here we hypothesize this may represent scenarios under which high resource density, increased landscape heterogeneity, or some unmeasured form of spatiotemporal disturbance would impact one or both species abundance [45]. The outlying nature of this point could plausibly have been derived from land conversion and logging, affecting predator abundance in Ulu Segama region at the time of data collection resulting in high co-abundance of felid species in our model. The initiation of the 2007–2009 forest management plan that saw a spike in removed vegetation and planting/rehabilitation efforts may explain this datum point as an outlier in our study. However, further research would be needed to confirm how such management practices impact both individual species and/or ecosystem networks. Thus, our findings prompt the question of whether the mechanism resulting in the parabolic relationship between abundances of clouded leopards and leopard cats is solely due to mesopredator release or whether, at least to some extent, it is due to a shift in carrying capacity, population structure, or other factors that contribute to changes in abundances that could also contribute to the relationship between these species across their spatiotemporal range.

Presuming, subject to further confirmatory research, that mesopredator release is one of the casual mechanisms of the species relationships we identify in our piecewise SEM, we propose the following hypothesis as to how mesopredator release might affect our study species. We speculate that leopard cats, in the absence of clouded leopards, either directly prey on terrestrial birds and/or create a landscape of fear altering the relative fecundity of prey [46]. However, crested fireback pheasants unlike the other prey species showed no significant response to either forest integrity or increases in mesopredator abundance, which we hypothesize is evidence of a non-uniform response to mesopredator abundance from prey species in this study [11]. It is possible that specific life-history traits or average prey body weight, insofar as this probably affects handling capacity of predators, determines response to the impact of increased leopard cat abundance [47]. Alternatively, the release of mesopredators might not be uniform across leopard cat demographics resulting in older/larger individuals persisting longer. In a parallel case involving domestic cats in Australia anecdotal evidence suggested these traits permit predation of “dangerous prey” including brushtailed possum (*Trichosurus vulpecula*), black-headed monitor (*Varanus tristis*), and domestic chicken (*Gallus gallus domesticus*) [48].

Among the mesopredators for which sample sizes in a camera-trapping study were too small to include in this analysis, the Bornean bay cat (*Catopmua badia*) and marbled cat (*Pardofelis marmorata*) could help further explain how the loss of clouded leopards impacts the interaction between the mesopredator and avifaunal communities. The bay cat displays similar temporal activity to those of birds, suggesting it may be a terrestrial bird specialist [26]. Novel methodology such as Bayesian co-abundance modeling, combined with both camera trapping and audio detection equipment, may provide future opportunities to investigate these more cryptic community interactions, especially if they can be incorporated into community models such as ours [49]. Broadly, alongside the global focus on invasive domestic cats as a major detriment to native fauna, we emphasize the parallel phenomenon whereby even native mesopredator species may have broad impacts on ecosystems in the absence of apex predators [50].

The use of forest integrity, as the single broad environmental predictor driving shifts in community dynamics, allowed us to account for fine-scale continuous change that any number of available categorical variables could not have done. As a holistic measure of deviation from the “natural forest state,” forest integrity allowed our model to account for both the observed and inferred ecological effects of forest loss without overburdening or distorting our results [29]. While our investigation of forest integrity matched closely our experience in the field, we are mindful that the dates of our data collection, and the creation of the forest integrity index in 2019, are not a perfect match in the context of the rapid rate of forest change in the region [4,22]. However, despite its shortcomings, we believe forest integrity remains a useful proxy for the overall, and possibly indirect, impact of anthropogenic activities and landscape changes on the ecosystem [51].

In highlighting potential mesopredator release, we acknowledge that the complexity of this interaction, and the underlying mechanics, necessitate further research. Furthermore, the use of piecewise SEMs, while powerful for building broad ecosystem-level snapshots, is still a relatively new method, that also merits further investigation and improvements. Nonetheless, our analysis provides insight into an ecosystem at a broad scale, highlighting previously unsuspected relationships and hopefully motivating the deeper exploration required to understand a system of this complexity.

## Funding

These analyses are based on camera-trapping surveys principally funded by the 10.13039/501100023278Darwin Initiative, Recanati-Kaplan Foundation, 10.13039/100013961Robertson Foundation, and 10.13039/100019524Sime Darby Foundation, with additional funding from the Clouded Leopard Project, the Felidae Conservation Fund, Houston Zoo, HG Wills International Trust for Nature Conservation, Panthera, the Dr. Holly Reed Conservation Fund of Point Defiance Zoo and Aquarium, and Wild About Cats. Publication was funded by Høgskolen i Innlandet (Inland Norway University of Applied Science).

## Ethics statements

The Economic Planning Unit of Malaysia, Sabah Biodiversity Council, Sabah Parks, Sabah Forestry Department, Sabah Wildlife Department and Yayasan Sabah reviewed all sampling procedures and approved permits for the work conducted. We applied non-invasive methods for data gathering and hence approval from an Institutional Animal Care and Use Committee or equivalent animal ethics committee was not required.

## Data statement

The data that has been used is confidential. Due to the sensitive nature of the species included in this research and the potential threat poaching poses to their populations, the raw data for this paper is publicly unavailable. Collaborative inquiries regarding data access may be possible at the discretion of the WildCRU team.

## CRediT authorship contribution statement

**Darwin S. Mayhew:** Writing – review & editing, Writing – original draft, Visualization, Software, Methodology, Formal analysis, Conceptualization. **Andrew J. Hearn:** Writing – review & editing, Project administration, Methodology, Investigation, Funding acquisition, Data curation. **Olivier Devineau:** Writing – review & editing, Validation, Software. **John D.C. Linnell:** Writing – review & editing, Supervision. **David W. Macdonald:** Writing – review & editing, Supervision, Project administration, Funding acquisition.

## Declaration of competing interest

The authors declare that they have no known competing financial interests or personal relationships that could have appeared to influence the work reported in this paper.The following represent public funding sources from charitable foundations or government agencies:

Andrew J. Hearn reports financial support was provided by Darwin Initiative.

David W. Macdonald reports financial support was provided by Recanati-Kaplan Foundation.

David W. Macdonald reports financial support was provided by Robertson Foundation.

Andrew J. Hearn reports financial support was provided by Sime Darby Foundation.

Andrew J. Hearn reports financial support was provided by Clouded Leopard Project.

Andrew J. Hearn reports was provided by Felidae Conservation Fund.

Andrew J. Hearn reports financial support was provided by Houston Zoo.

David W. Macdonald reports financial support was provided by HG Wills International Trust for Nature Conservation.

David W. Macdonald reports financial support was provided by Panthera Corp.

Andrew J. Hearn reports financial support was provided by the Dr. Holly Reed Conservation Fund of Point Defiance Zoo and Aquarium.

Andrew J. Hearn reports financial support was provided by Wild About Cats.

We thank Danum Valley Management Committee, Sabah Parks, Sabah Forestry Department, Sabah Wildlife Department, Yayasan Sabah, the Economic Planning Unit and the Sabah Biodiversity Centre for permission to conduct research. We thank Sam Cushman, Carol Sartor, and Morten Odden for insightful comments.

The Economic Planning Unit of Malaysia, Sabah Biodiversity Council, Sabah Parks, Sabah Forestry Department, Sabah Wildlife Department and Yayasan Sabah reviewed all sampling procedures and approved permits for the work conducted. We applied non-invasive methods for data gathering and hence approval from an Institutional Animal Care and Use Committee or equivalent animal ethics committee was not required. If there are other authors, they declare that they have no known competing financial interests or personal relationships that could have appeared to influence the work reported in this paper.

## Figures and Tables

**Table A1:** ^b^

Grid Name		Number Cameras	Collection Dates	Number of Sub-grids	Number Camera in Sub-grids
Crocker		35	June 6th, 2011 to Feb. 7th, 2012	2	18, 17
Danum Palm		23	March 10th, 2009 to July 8th, 2009	1	23
Danum Valley		79	Feb. 24th^,^ 2012 to Oct. 6th, 2012	5	16, 17, 15, 15, 16
IJM		33	May 25th, 2011 to Aug. 18th, 2011	2	19, 14*
Sepilok		35	Feb. 9th, 2011 to May 24th, 2011	2	20, 15*
Kinabatangan		66	July 25th, 2010 to Dec. 17th, 2010	4	18, 16, 15, 17
Malua		38	July 11th, 2008 to Feb. 11th, 2009	2	17, 21
Tabin		75	Aug. 19th, 2009 to April 26th, 2010	5	15, 15, 15, 15, 15
Tawau		77	Oct. 31st, 2012 to Jan 18th, 2014	5	15, 15, 16, 15, 16
Sabah Softwoods	15	March 25th, 2021 to July 11th, 2021	1	15*	
Ulu Segama		22	May 25th, 2007 to Oct. 17th, 2007	1	22
All		498	May 25th, 2007 to July 11th, 2021	30	Minimum of fifteen

*Two traps stolen or broken from this sub-grid.

^b^ Camera trapping grids were named after their relative areas with varying dates of collection. The number of cameras also varied as available areas to survey and funding varied. Sub-grids were randomly selected using K-mean clustering with equal sized clusters developed by Bradley et al. [28]. Camera grids were broken into sub-grids with a minimum of at least fifteen cameras at each to give most sub-grids somewhat equal camera numbers relative to the smallest camera grids while optimizing the total number of sub-grids possible from each grid. Four camera locations were removed post subdivision as they were stolen or broken while in the field resulting in no data being collected.

**Table A2:** ^c^

Camera Grid	Sub-grid	Sub-grid Size	Mean Forest Integrity	Mean Forest Integrity Standard Deviation	Sunda Clouded Leopard	Sunda Clouded Leopard Standard Error	Sunda Leopard Cat	Sunda Leopard Cat Standard Error	Great Argus Pheasant	Great Argus Pheasant Standard Error	Bulwer's Pheasant	Bulwer's Pheasant Standard Error	Crested Fireback	Crested Fireback Standard Error	Pitta Family	Pitta Family Standard Error
Crocker	Crocker 1	18	916	85	113	37	97	34	193	42	199	46	166	73	84	30
Crocker	Crocker 2	17	839	158	61	28	58	29	489	76	35	20	0	0	31	18
Danum Palm	Danum Palm	17	596	406	0	0	1775	174	28	14	0	0	3005	538	0	0
Danum Valley	Danum Valley 1	16	986	4	204	58	66	30	415	67	302	65	339	105	131	45
Danum Valley	Danum Valley 2	17	994	1	87	42	18	19	445	78	234	63	1007	173	79	35
Danum Valley	Danum Valley 3	15	979	32	203	91	56	40	1393	162	225	77	846	170	99	48
Danum Valley	Danum Valley 4	15	943	42	148	74	174	72	1176	140	232	70	402	83	37	22
Danum Valley	Danum Valley 5	16	978	28	237	83	29	28	1159	145	128	55	1130	222	88	40
IJM	IJM 1	19	270	173	0	0	648	158	0	0	0	0	855	389	0	0
IJM	IJM 2	16	184	222	0	0	1259	305	0	0	0	0	0	0	0	0
Kinabatangan	Kinabatangan 1	18	387	226	27	28	53	31	0	0	0	0	552	127	10	10
Kinabatangan	Kinabatangan 2	16	574	275	26	26	91	53	13	13	0	0	1189	224	12	12
Kinabatangan	Kinabatangan 3	15	404	158	272	120	24	24	0	0	0	0	337	86	26	19
Kinabatangan	Kinabatangan 4	17	669	214	66	46	22	22	127	42	0	0	902	197	0	0
Malua	Malua 1	17	932	32	27	20	201	48	0	0	0	0	178	76	9	9
Malua	Malua 2	21	948	59	58	29	127	31	0	0	0	0	86	37	0	0
Sepilok	Sepilok 1	20	664	124	0	0	38	27	254	54	0	0	117	69	29	17
Sepilok	Sepilok 2	15	689	137	0	0	113	58	469	90	0	0	0	0	0	0
Tabin	Tabin 1	15	927	33	128	53	91	42	595	88	0	0	308	65	11	11
Tabin	Tabin 2	15	945	17	106	45	147	48	342	79	0	0	546	114	0	0
Tabin	Tabin 3	15	894	48	166	68	236	75	335	71	0	0	504	89	0	0
Tabin	Tabin 4	15	742	138	89	46	175	64	265	58	0	0	601	103	11	11
Tabin	Tabin 5	15	858	54	122	68	18	18	105	36	0	0	350	69	20	15
Sabah Softwoods	Sabah Softwoods	17	28	69	0	0	664	159	0	0	0	0	0	0	0	0
Tawau	Tawau 1	15	754	214	132	37	110	34	892	91	186	40	349	106	51	20
Tawau	Tawau 2	15	900	7	184	56	44	22	266	47	429	69	354	118	39	17
Tawau	Tawau 3	16	862	28	100	32	59	26	525	68	96	30	19	15	0	0
Tawau	Tawau 4	15	891	27	118	50	40	23	236	51	0	0	324	63	33	19
Tawau	Tawau 5	16	918	5	168	50	71	25	712	77	445	70	0	0	14	10
Ulu Segama	Ulu Segama	17	916	72	362	110	334	63	223	76	0	0	609	100	0	0

^c^ Modified abundance (±SE) measurements derived from the Royle-Nichols and N-mixture abundance models, multiplied by 100, and rounded to the nearest integer for each focal species in each study site. Camera grids are listed by alphabetical order in addition to sub-grids and sub-grid sizes, forest loss integrity index is the mean (±SD) of each grid multiplied by 100 and rounded to the nearest integer on a scale from 0 (Low) to 1000 (High) based on Grantham et al. [29].

**Table A3:** ^d^

Species	Detection	Abundance	Number of Parameters	AICc	ΔAICc	AICc Weight
Sunda Clouded Leopard: Royle-Nichols						
	∼ Effort + Forest Road	∼ Camera Sub-grid	34	1712.81	0.00	0.78
	∼1	∼1	2	1883.62	170.82	6.3e-38
Sunda leopard cat: Royle-Nichols						
	∼ Effort + Forest Road + Ridge	∼ Camera Sub-grid	34	1838.51	0.00	0.54
	∼ Effort + Forest Road	∼ Camera Sub-grid	33	1838.81	0.29	0.46
	∼1	∼1	2	2327.40	488.88	3.7e-107
Great Argus Pheasant: N-Mixture						
	∼ Effort + Forest Road	∼ Camera Sub-grid	33	6107.90	0.00	0.54
	∼ Effort + Forest Road + Ridge	∼ Camera Sub-grid	34	6108.25	0.34	0.46
	∼1	∼1	2	7210.91	1103.01	1.7e-240
Bulwer's Pheasant: N-Mixture						
	∼ Effort	∼ Camera Sub-grid	32	1805.21	0.00	0.58
	∼ Effort + Ridge	∼ Camera Sub-grid	33	1805.82	0.61	0.42
	∼1	∼1	2	2268.23	463.02	1.6e-101
Crested Fireback: N-Mixture						
	∼ Effort + Ridge	∼ Camera Sub-grid	34	3332.29	0.00	0.64
	∼ Effort + Forest Road + Ridge	∼ Camera Sub-grid	35	3333.47	1.18	0.36
	∼1	∼1	3	3808.46	476.17	2.6e-104
Pitta Family: Royle-Nichols						
	∼ Effort	∼ Camera Sub-grid	32	697.86	0.00	0.46
	∼ Effort + Ridge	∼ Camera Sub-grid	33	698.95	1.09	0.27
	∼1	∼1	2	755.25	57.39	1.6e-13

^d^ Abundance model selection using both the Royle-Nichols model of abundance for Sunda clouded leopard, Sunda leopard cat, and the Pitta family, and N-mixture model of abundance for great argus pheasant, Bulwer's pheasant, and crested fireback. Forest road and ridge, short for ridge line, are both binary covariates that indicate if a camera was or was not placed on one or both landscape structures. Both model types estimate abundance as well as detection probability. We present models for each species with ΔAIC less than 2, as well as the null model.

**Table A4:** ^e^

Dependent Variable	Independent Variable	Test Type	Degrees of Freedom	Critical Value	P-Value
Sunda Leopard Cat	Forest Integrity	Coefficient	26	−0.7260	0.4743
Great Argus Pheasant	I (Sunda Clouded Leopard)	Coefficient	26	−1.4646	0.1550
Pitta Family	I (Sunda Clouded Leopard)	Coefficient	26	0.6295	0.5345
Sunda Clouded Leopard	Crested Fireback	Coefficient	27	−1.2745	0.2133
Bulwer's Pheasant	Crested Fireback	Coefficient	24	−1.4252	0.1670
Sunda Leopard Cat	Crested Fireback	Coefficient	26	1.1208	0.2726
Great Argus Pheasant	Crested Fireback	Coefficient	26	−0.2442	0.8090
Pitta Family	Crested Fireback	Coefficient	26	0.2903	0.7739
Great Argus Pheasant	Sunda Clouded Leopard	Coefficient	26	−1.6125	0.1189
Pitta Family	Sunda Clouded Leopard	Coefficient	26	1.2284	0.2303
Great Argus Pheasant	Bulwer's Pheasant	Coefficient	24	0.9852	0.3343
Pitta Family	Great Argus Pheasant	Coefficient	26	1.5520	0.1327

^f^ List of independence claims as the results from the test of d-separation run during the evaluation of our finial piecewise SEM model. Significant relationships would be indicated with an asterix however, no significant relationships remain after specifying the relationship between the pitta family and Bulwer's pheasant along with Sunda clouded leopards and the squared values of Sunda clouded leopards as correlated errors.

**Table A5:** ^f^

Response Variable		Predictor Variable	Model Type
Sunda Clouded Leopard Abundance (CL Abund)	Mean Sub-grid Forest Integrity	GLM (Negative Binomial distribution)	
Sunda Leopard Cat Abundance (LC Abund)	CL Abund + I (CL Abund^2)	GLM (Negative Binomial distribution)	
Great Argus Pheasant	LC Abund +Mean Sub-grid Forest Integrity	GLM (Negative Binomial distribution)	
Bulwer's Pheasant	CL Abund + I (CL Abund^2) + LC Abund +Mean Sub-grid Forest Integrity	GLM (Negative Binomial distribution)	
Crested Fireback		∼1	N/A
Pitta Family	LC Abund +Mean Sub-grid Forest Integrity	GLM (Negative Binomial distribution)	
Sunda Clouded Leopard/I (Sunda Clouded Leopard)		Sunda Clouded Leopard/I (Sunda Clouded Leopard)	Correlated Error
Bulwer's Pheasant/Pitta Family	Bulwer's Pheasant/Pitta Family	Correlated Error	

^e^ Model structures of pathways that compose the final piecewise structural equation model. Model types were restricted to general linear models only as the sample size was too small for mixed effect models to be used. These models represent the most parsimonious models for our data given our limited flexibility.
